# Older adults’ reporting of specific sedentary behaviors: validity and reliability

**DOI:** 10.1186/1471-2458-14-734

**Published:** 2014-07-21

**Authors:** Jelle Van Cauwenberg, Veerle Van Holle, Ilse De Bourdeaudhuij, Neville Owen, Benedicte Deforche

**Affiliations:** 1 Department of Human Biometry and Biomechanics, Faculty of Physical Education and Physical Therapy, Vrije Universiteit Brussel, Pleinlaan 2, 1050 Brussels, Belgium; 2 Department of Movement and Sport Sciences, Faculty of Medicine and Health Sciences, Ghent University, Watersportlaan 2, 9000 Ghent, Belgium; 3 Fund for Scientific Research Flanders (FWO), Egmontstraat 5, 1000 Brussels, Belgium; 4 Baker IDI Heart and Diabetes Institute, The University of Queensland, Melbourne University and Monash University, Commercial Rd, Melbourne 3004Victoria, Australia

**Keywords:** Sitting time, Television viewing, Psychometrics, Seniors, Self-report, Accelerometers

## Abstract

**Background:**

Previous questionnaires targeting older adults’ sedentary time have underestimated total sedentary time, possibly by not including all relevant specific sedentary behaviors. The current study aimed to investigate the criterion validity and test-retest reliability of a new questionnaire assessing a comprehensive set of sedentary behaviors. Additionally, we examined whether the criterion validity of the questionnaire differed according to age, gender and educational level.

**Methods:**

A sample of home-dwelling Belgian older adults (>64 years, n = 508) completed a newly-developed questionnaire assessing twelve specific sedentary behaviors and wore an accelerometer for seven consecutive days as criterion measure. A subsample (n = 28) completed the questionnaire a second time to examine test-retest reliability. Data collection occurred between September 2010 and October 2012.

**Results:**

Correlational analyses examining self-reported total sitting time and accelerometer-derived sedentary time yielded a Spearman’s ρ of 0.30. Using the Bland-Altman regression procedure, self-reported total sitting time underestimated accelerometer-derived sedentary time by -82 minutes/day for a participant with an average level of sedentary time (539 minutes/day). Corresponding 95% limits of agreement were wide (-364, 200 minutes/day). Better, but still not ideal, validity findings were observed in the younger, male and tertiary-educated subgroups. Acceptable test-retest reliability (ICC > 0.70) was found for total sitting time, TV viewing, computer use, and driving a car.

**Conclusion:**

Validity for older adults’ self-reported total sitting time against accelerometer-derived sedentary time was not strong, but comparable to previous studies. However, underestimation of total sedentary time was lower compared to previous studies, possibly explained by the inclusion of additional specific sedentary behaviors. Further research is needed to develop self-report tools and objective criterion measures that accurately measure engagement in (specific) sedentary behavior(s) among different subgroups of the older population.

## Background

Sedentary behaviors, defined prolonged sitting and low levels (1.0-1.5 METs) of energy expenditure [1] are associated with morbidity and premature mortality, additional to the influence of moderate-to-vigorous physical activity [2-8]. Older adults (≥65 years) are the most sedentary age group with average levels of objectively assessed sedentary time reaching 540 minutes/day and more [9]. Total sedentary time can encompass several specific behaviors (e.g., television viewing, motorized transport, reading, computer use). Different sedentary behaviors have been linked to different health outcomes [10,11] and reducing them will require particular intervention strategies in different contexts [12]. While accelerometer measurement provides an objective assessment of older adults’ total sedentary time, questionnaires are needed to assess engagement in specific sedentary behaviors [13].

In their review on measures of sedentary behaviors in adults (≥18 years), Clark et al. [14] concluded that there is a lack of reliable and valid questionnaires covering a wide range of specific sedentary behaviors. In older adults, only two studies have examined the measurement properties of a sedentary behavior questionnaire that included multiple questions on engagement in multiple specific sedentary behaviors rather than one question targeting participants’ overall sitting time [15,16]. In both studies, time spent in the specific sedentary behaviors was summed to obtain a measure of total sitting time, which was then validated against accelerometer-derived sedentary time. These self-report measures did not exhibit strong validity, with self-reported total sitting time underestimating accelerometer-derived sedentary time on average by 216 minutes/day [15] and 406 minutes/day [16]. The authors concluded that this underestimation might have partially resulted from the questionnaires not including some specific sedentary behaviors, such as time spent eating, sitting while telephoning, and sitting during household chores. Only one of those studies [15] reported separately on the reliability of multiple specific sedentary behaviors.

Previous studies have reported differences between men and women in validity findings for a sedentary behavior questionnaire, in US overweight adults [17] and in European adolescents [18]. Next to gender, other demographic factors, such as age and educational level, may also influence validity. Except for one study testing the reliability and validity of a question targeting overall sitting time among residents of low versus high socio-economic neighborhoods of Hong Kong [19], differences in validity results of a sedentary behavior questionnaire between demographic subgroups of the older population remain unexplored.

There is a need for the development and examination of the measurement properties of self-report instruments to address the broader range of older adults’ sedentary behaviors. We examined the criterion validity and test-retest reliability of a new questionnaire assessing a comprehensive set of specific sedentary behaviors in older adults. Additionally, we examined whether the criterion validity of the questionnaire differed according to age, gender and educational level.

## Methods

### Procedures

Contact details and age of all older adults (≥65 years) residing in Ghent (Flanders, Belgium) were obtained from the city’s public service department. We selected 1,750 potential participants through random sampling stratified by age (65–74 vs. ≥ 75 years) and gender. They were sent a letter that explained the study protocol and informed them that a researcher would visit them within the next 14 days to ascertain their willingness (or otherwise) to participate. The researcher made three attempts to find the potential participant at home.

Following agreement to participate, the protocol was explained in full, an informed consent form was signed, and data collection was started. For inclusion, participants had to be non-institutionalized and not limited by their health to walk a couple of 100 meters. The latter criterion was derived from an item included in the SF-36, the most frequently used questionnaire to assess health status and quality of life [20,21]. Participants were asked whether and to what degree they were limited by their health to walk a couple of 100 meters. Response categories are: (1) yes, seriously limited, (2) yes, somewhat limited, and (3) no, not limited. Those who reported being seriously or somewhat limited were excluded from participation. In total, 1,260 older adults were found at home, of which 627 (49.8%) were not willing to participate and 125 (9.9%) were classified as not eligible. This resulted in 508 participating in the study, a response rate of 44.8% (508/1,135 eligible participants found at home). Data were collected between September 2010 and October 2012. The study protocol was approved by the Ghent University Hospital.

The study protocol was completed in two home visits. During the first visit a structured interview that assessed health status, physical activity and sedentary behaviors was conducted. The participant was also provided with an accelerometer to wear during the next seven days and an appointment for a second home visit approximately 8 days later was made. Additionally, participants were randomly selected by the researcher (stratified by gender) and asked whether they were willing to answer an additional questionnaire during the second home visit. During the second home visit a structured interview assessed demographic factors, anthropometric measures (weight and height) were performed and the accelerometer was collected. In a subsample of 28 participants who agreed to answer the additional questions (response rate not recorded), the same questionnaire targeting engagement in different sedentary behaviors was administered for the second time to assess its test-retest reliability. Mean time between test and retest of the sedentary behavior questionnaire was 9.6 (±1.7) days.

### Measures

#### Socio-demographic factors and health status

Socio-demographic factors were assessed: age, gender, marital status, educational level, and (former) occupation. Age was dichotomized as 65–74 years and 75+ years old. Educational level was assessed using a 6-point scale ranging from having completed primary to university education. This was dichotomized as non-tertiary and tertiary (including college and university) education. The SF-36 [20] was used to assess health status and functional limitations. To calculate body mass index (BMI), height and weight were measured with a SECA 214 stadiometer and a SECA 813 Robusta weight scale up to 0.1 cm and 0.1 kg, respectively.

#### Self-reported sedentary behaviors

For the current study, a new sedentary behavior questionnaire was developed (see Additional file 1). We aimed to develop a questionnaire that was easy to administer and that covered a wide range of sedentary behaviors relevant to older adults. Since many studies include measures of both physical activity and sedentary behaviors, using a similar format for both measures would facilitate comprehension and ease of administration. The International Physical Activity Questionnaire (IPAQ, available at http://www.ipaq.ki.se) is a frequently used tool to assess physical activity [22], and, therefore, we chose the format of our sedentary behavior questionnaire to be similar to that of the IPAQ. In the current study, a version of the IPAQ, specifically adapted for administration among Flemish older adults, was completed prior to the sedentary behavior questionnaire. This version of the IPAQ only included questions targeting physical activity behaviors and did not include questions targeting sitting time. Similar to the IPAQ, the new sedentary behavior questionnaire uses open-ended response options that avoid possible ceiling effects observed in sedentary behavior questionnaires with closed response options [16]. We used the ‘last seven days’ as target period because it was considered that this would be easier to recall accurately than would the ‘usual week’. Furthermore, the ‘last seven days’ timeframe is the most frequently used time frame of the IPAQ [22] and it was preferred over the ‘usual week’ by most study sites in a 12-country validity and reliability study of the IPAQ [23]. An interview format was used to provide a more standardized administration than would be achieved using self-administered questionnaires [24]. To include a wide range of sedentary behaviors, we combined the sedentary behaviors included in previous questionnaires [15,25,26], and complemented these with additional sedentary behaviors relevant for older adults. More specifically, we subdivided questions targeting sitting in a car into driving a car, being a car passenger and using public transport. We added one question targeting usual time spent sitting while eating in the last seven days (in minutes/day) as was suggested by Gardiner et al. [15]. Furthermore, we added questions targeting sitting while doing household chores (e.g., ironing, preparing a meal). Except for usual time spent sitting while eating, all specific sedentary behaviors were assessed with two open-ended questions. Similar to the IPAQ, a first question assessed on how many days the behavior was performed in the last seven days, while the second question prompted how long, on average, the participant engaged in that sedentary behavior on such a day. Since eating can be expected to occur on a daily basis, sitting while eating was assessed with one question targeting the usual time spent sitting while eating in the last seven days. In total, the following 12 sedentary behaviors were included: TV viewing, computer use, reading, sedentary hobbies (e.g. handicraft, playing cards), having a seated conversation or listening to music, telephone use, public transport, driving a car, being passenger in a car, sitting during household chores, resting, and eating. The new questionnaire was pilot-tested in a convenience sample (n = 4) of community-dwelling Flemish older adults to assess older adults’ understanding and completeness of the different items. Researchers involved in data collection were explicitly trained to ensure participants reported sedentary behaviors in which they engaged during the last seven days and did not duplicate their reported sedentary times across different sedentary items.

The average daily time spent in the different sedentary behaviors was calculated as follows: (number of days engaged in the behavior * average time engaged in the behavior on such a day)/7. The average daily times spent in the different sedentary behaviors were summed to create the variable ‘self-reported total sitting time’. Participants with self-reported total sitting times higher than 18 h/day (n = 7) were excluded.

#### Accelerometer-derived sedentary time

The Actigraph GT3X + accelerometer served as criterion measure of overall sedentary time. Actigraph accelerometers are the most frequently used tools to measure physical activity and sedentary behavior in population-based studies among older adults [27]. These accelerometers register accelerations of the human body; their output (counts/minute) can be used to derive the intensity at which activities were performed. However, this type of accelerometer cannot distinguish between different postures; they cannot distinguish whether registered counts originated from lying, sitting or standing activities [28]. Other types of devices, such as the activPAL, measure thigh inclination from which posture (lying, sitting, or standing) can be inferred [29]. However, their use is less common in population-based studies [9,27]. Furthermore, Healy et al. [9] have shown that Actigraph accelerometer-derived sedentary time has minimal bias compared to activPAL-derived sedentary time.

Participants wore an Actigraph GT3X + accelerometer during seven consecutive days. Accelerometers were initialized to start registration on the morning after the first home visit. Participants were asked to wear the accelerometer on the right hip during waking hours, but to remove the device during bathing activities or contact sports. Accelerometers were initialized and data downloaded using Actigraph version 6.0, data were cleaned using Meterplus version 4.3. Data registration occurred in 1 min epochs. Twenty-eight participants had no accelerometer data due to device-failure. As recommended by Choi et al. [30], a period of at least 90 minutes of consecutive zeros was defined as non-wear time. A valid day was defined as a day that contained at least 10 hours of accelerometer data and participants with less than five valid days were excluded from further analyses [19,31]. Based on this, 25 participants were excluded. Participants with more than 18 valid hours/day were also excluded (n = 6). This resulted in the inclusion of 442 participants with complete questionnaire and accelerometer data with a mean of 15.0 ± 1.4 valid hours/valid day. Minutes with less than 100 activity counts were defined as sedentary minutes [32]. Moderate-to-vigorous physical activity (MVPA) was defined as ≥ 1952 counts per minute [33].

### Statistical analyses

All analyses were performed using IBM SPSS Statistics version 20. Significance level was defined at 0.05. For the criterion validity analysis, the total analytic sample included 442 participants. To analyze the criterion validity, a Spearman rank correlation coefficient between self-reported total sitting time (as assessed during the second home visit) and accelerometer-derived sedentary time was calculated. For physical activity questionnaires, Terwee et al. [34] proposed to use a threshold of 0.50 to define a measure of self-reported total physical activity as valid against accelerometer counts. To assess absolute agreement between the two measures, the Bland-Altman regression procedure was followed [35]. During this procedure, a simple linear regression analysis is performed between the average of self-reported total sitting time and accelerometer-derived sedentary time and the difference between these two measurements. The plot of this regression analysis (a Bland-Altman plot), that includes the trend line with 95% limits of agreement, was used to illustrate the absolute agreement between the two measures. To examine the criterion validity in different demographic subgroups, this procedure was repeated for subgroups based on age, gender and educational level.

To assess test-retest reliability between the two self-report measurements of the 12 specific sedentary behaviors and total sitting time, single-measures intraclass correlation coefficients (ICC) with corresponding 95% confidence intervals were calculated using two-way mixed-effects models. Test-retest reliability was considered acceptable when the corresponding ICC ≥ 0.70 [34,36].

## Results

### Sample characteristics

Table 1 presents the descriptive characteristics and daily minutes of engagement in (specific) sedentary behaviors of the sample and subsample used for the reliability analysis. Participants from the subsample were slightly older, were more likely to have followed tertiary education and performed a white collar job, rated their health better, were less limited to walk, accumulated more accelerometer-derived sedentary time, but reported less total sitting time compared to participants from the total sample.

**Table 1:** Descriptive characteristics and daily minutes of sedentary behavior(s) of the total analytic sample and subsample

**Characteristics**	**Total analytic sample (n = 442)**	**Subsample**^**a **^**(n = 28)**
Age (M ± SD)	74.2 ± 6.2	76.6 ± 6.5
Gender (% women)	54.8	50.0
Marital status (%)		
Widowed	21.2	21.4
Never married/divorced	12.7	10.7
Married/cohabiting	66.1	67.9
Educational level (%)		
Primary education	25.8	18.5
Secondary education	36.1	29.6
Tertiary education	38.1	51.8
Occupation (%)		
Household	18.0	14.3
Blue collar	26.9	21.5
White collar	55.1	64.3
BMI (M ± SD)	22.3 ± 3.6	22.4 ± 2.9
Self-rated health (% fair/poor)^b^	18.1	14.3
% limited to walk more than 1 km^b^	26.5	28.6
Accelerometer-derived MVPA (M ± SD)	16.2 ± 16.8	17.0 ± 11.4
Accelerometer-derived sedentary time (M ± SD)	580.4 ± 97.7	596.5 ± 111.5
TV viewing (Med; Q1-Q3)	175.7; 90.0 - 240.0	175.7; 60.0 – 180.0
Computer use (Med; Q1-Q3)	0.0; 0.0 - 60.0	0.0; 0.0 – 93.8
Reading (Med; Q1-Q3)	60.0; 30.0 – 93.2	60.0; 30.0 – 120.0
Sedentary hobbies (Med; Q1-Q3)	0.0; 0.0 – 34.3	0.0; 0.0 – 25.7
Seated conversation or listening to music (Med; Q1-Q3)	25.7; 5.4 – 51.4	25.7; 4.3 – 51;4
Telephone use (Med; Q1-Q3)	1.4; 0.0 – 8.6	1.1; 0.0 – 20.0
Public transport (Med; Q1-Q3)	1.4; 0.0 – 11.4	11.1; 0.0 – 25.7
Driving a car (Med; Q1-Q3)	8.6; 0.0 - 22.9	0.0; 0.0 – 23.6
Passenger in a car (Med; Q1-Q3)	0.0; 0.0 - 8.6	3.6; 0.0 – 12.9
Sitting during household chores (Med; Q1-Q3)	0.0; 0.0 - 0.0	0.0; 0.0 - 0.0
Resting (Med; Q1-Q3)	19.3; 0.0 – 45.0	10.7; 0.0 – 43.8
Eating (Med; Q1-Q3)	75.0; 60.0 – 90.0	90.0; 60.0 – 90.0
Total sitting time (Med; Q1-Q3)	475.0; 383.0 - 599.0	459.6; 356.3 – 585.2

^a^Subsample used for the reliability analysis.

^b^Derived from the SF-36 questionnaire.

M = mean, SD = standard deviation, Med = median, Q1 = quartile 1, Q3 = quartile 3.

All physical activity and sedentary behaviors are expressed in minutes/day.

### Criterion validity

**Figure 1:**
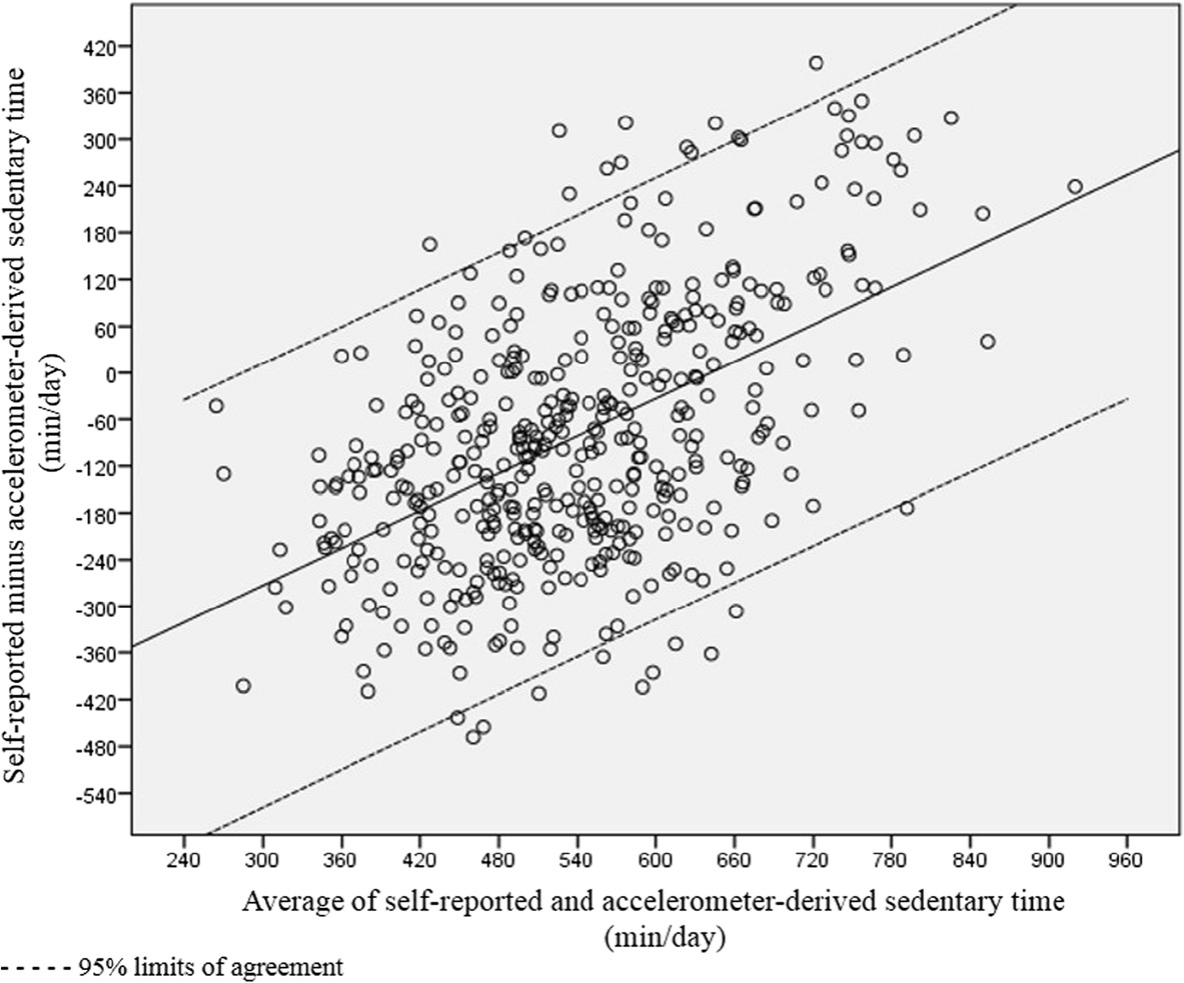
Bland-Altman plot for self-reported total sitting time and accelerometer-derived sedentary time in the total sample.

**Figure 2:**
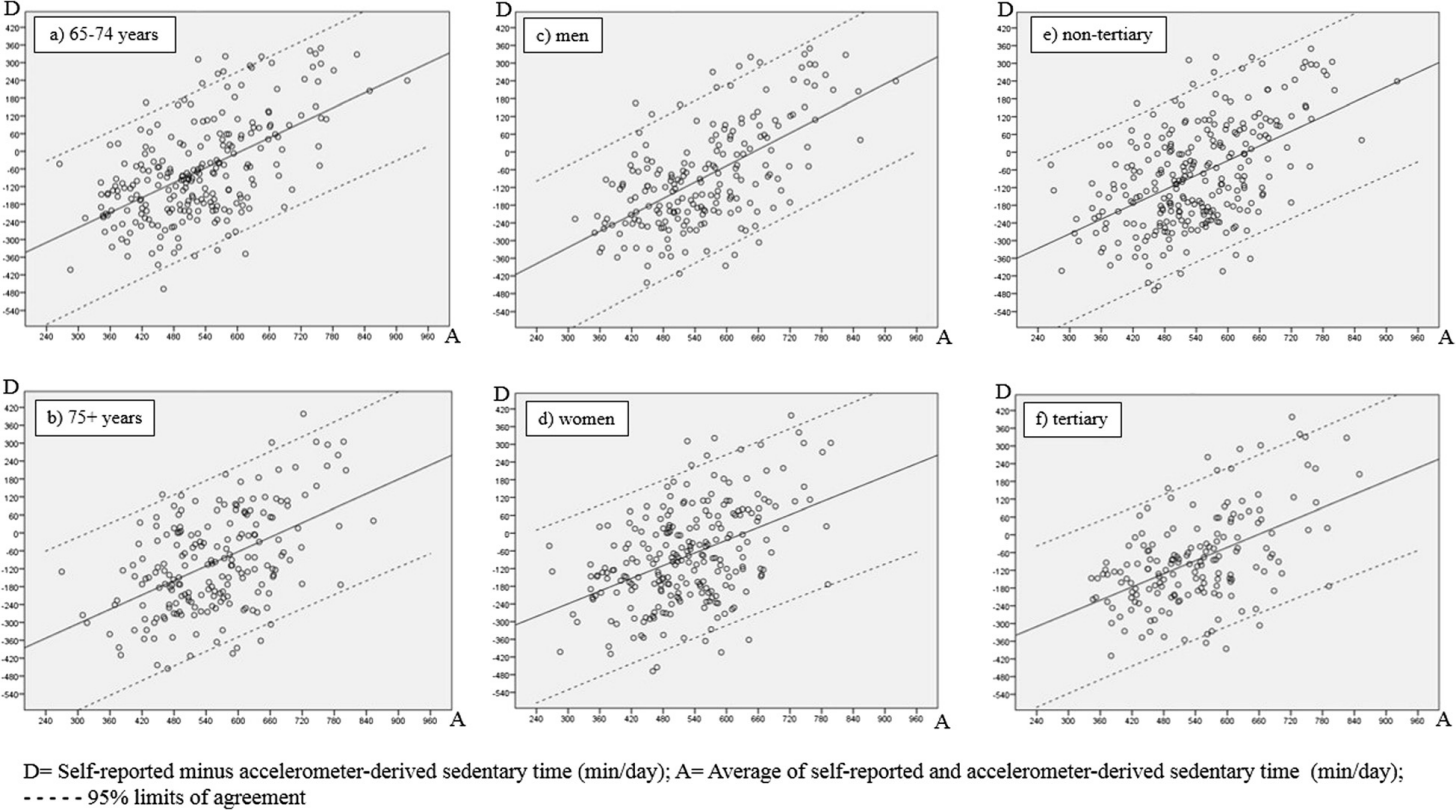
Bland-Altman plots for self-reported total sitting time and accelerometer-derived sedentary time in the 65- to 74-year-old (a), +75-year-old (b), male (c), female (d), non-tertiary (e) and tertiary educated (f) subgroups.

Results for the criterion validity analysis in the total sample and the subgroups based on age, gender and education are presented in Table 2. In the total sample, correlation analysis between self-reported total sitting time and accelerometer-derived sedentary time yielded a Spearman’s ρ of 0.30 (p < 0.001). Following the Bland-Altman regression procedure [35], a significant positive relationship was observed between the average of self-reported and accelerometer-derived measurements and the difference between these two measurements (B = 0.80, S.E. = 0.06, p < 0.001) (see Figure 1). The difference between self-reported and accelerometer-derived sedentary behaviors was estimated as -512.46 + (0.80* average of the two measurements). This yielded a mean difference of -81.88 minutes/day relative to the mean average of the two measurements (539.58 minutes/day). Corresponding 95% limits of agreement were wide (-364.16; 200.41 minutes/day), implying strong variability surrounding these general trends. For lower and medium averages of self-reported and accelerometer-derived sedentary time, self-reported total sitting time underestimated the accelerometer-derived measurement. For averages higher than 640 minutes/day, self-reported total sitting time overestimated the accelerometer-derived measurement.Similar patterns were observed in the subgroups based on age, gender and education; the average of self-reported and accelerometer-derived measurements was significantly positively related to the difference between these two measurements with underestimation at lower and medium averages and overestimation at higher averages (see Figure 2). However, in the 65- to 74-year-old, male and tertiary educated subgroups the correlation between self-reported and accelerometer-derived sedentary time was substantially stronger than in the older, female and non-tertiary educated subgroups. Furthermore, in the 65- to 74-year-old, male and tertiary educated subgroups, the standard deviations of the residuals (and correspondingly the 95% limits of agreement) were smaller.

**Table 2:** Validity results in the total sample and subgroups based on age, gender and education

	**Spearman’s rho**^**a**^	**Bland-Altman procedure**		
		**Regression equation**^**b**^**: D = b**_**0**_ **+ (b**_**1 **_**× A)**	**Standard deviation of the residuals**	**D at A = 540 minutes/day**^**c **^**(95% LOA)**
**Total sample**	0.30	-512.46 + (0.80 × A)	144.02	-81.88 (-364.16; 200.41)
**Age**				
65-74 years	0.35	-512.96 + (0.85 × A)	138.37	-53.96 (-325.17; 217.24)
75+ years	0.24	-546.49 + (0.81 × A)	144.25	-109.09 (-391.82; 173.64)
**Gender**				
Men	0.35	-599.96 + (0.92 × A)	138.68	-103.55 (-375.36; 168.26)
Women	0.24	-455.09 + (0.72 × A)	145.96	-66.59 (-352.67; 219.49)
**Education**				
Non-tertiary	0.25	-525.66 + (0.83 × A)	149.07	-77.46 (-369.64; 214.72)
Tertiary	0.39	-489.48 + (0.75 × A)	134.44	-84.48 (-347.98; 179.02)

D = difference between self-reported total sitting time and accelerometer-derived sedentary time; A = average of self-reported total sitting time and accelerometer-derived sedentary time; 95% LOA = 95% limits of agreement = D ± (1.96 × standard deviation of the residuals).

^a^All correlations were statistically significant at p < 0.001.

^b^All b_1_’s were statistically significant at p < 0.001.

^c^540 minutes/day is the mean of the average of self-reported total sitting time and accelerometer-derived sedentary time in the total sample.

### Test-retest reliability

Table 3 presents the results for the test-retest reliability analyses of the twelve self-reported specific sedentary behaviors and total sitting time. Acceptable test-retest reliability (ICC > 0.70) was found for TV viewing, computer use, driving a car, and total sitting time.

**Table 3:** Results of the reliability analysis

**Sedentary behavior items**	**ICC (95% C.I.)**
TV viewing	0.92 (0.83, 0.96)
Computer use	0.76 (0.54; 0.88)
Reading	0.60 (0.29; 0.79)
Sedentary hobbies	0.57 (0.26; 0.78)
Seated conversation or listening to music	0.40 (0.04; 0.67)
Telephone use	0.69 (0.43; 0.84)
Public transport	0.46 (0.11; 0.71)
Driving a car	0.79 (0.59; 0.90)
Passenger in a car	0.11 (-0.27; 0.46)
Sitting during household chores	0.12 (-0.26; 0.47)
Resting	0.20 (-0.18; 0.53)
Eating	0.46 (0.11; 0.71)
Total sitting time	0.77 (0.57; 0.89)

ICC = Intraclass Correlation Coefficients.

C.I. = Confidence Interval.

## Discussion and conclusions

We examined the validity of self-reported total sitting time relative to accelerometer-derived sedentary time among older adults and in different demographic subgroups. Validity was not strong, with a Spearman correlation of 0.30 in the total sample and wide limits of agreement. However, these relationships were stronger than those reported by Hekler et al. [16], who found a correlation of 0.12 among US older adults, and are comparable to the findings of Gardiner et al. [15], who reported a correlation of 0.30 among Australian older adults. Furthermore, with 82 minutes/day for a mean average of self-reported and accelerometer-derived sedentary time, our self-report measure of total sitting time underestimated accelerometer-derived sedentary time substantially less than the self-report measures used in the previous studies [15,16]. This apparently lower level of underestimation for a mean average of self-reported and accelerometer-derived sedentary time might be explained by the current questionnaire including specific sedentary behaviors that were not employed in previous questionnaires. Several explanations might account for the remaining average underestimation of 82 minutes/day. Firstly, participants might simply be unable to accurately recall and estimate durations of engagement in certain sedentary behaviors. Secondly, social desirability might have resulted in underreporting of certain sedentary behaviors (i.e. television viewing). Thirdly, accelerometers are not the ideal criterion measure to assess sedentary behavior as they cannot distinguish between different postures. Hence, accelerometers might have overestimated older adults’ sedentary time by classifying standing activities at very light intensities as sedentary. However, Healy et al. [9] identified accelerometer-derived sedentary time as having relatively minimal bias compared to activPAL-derived sedentary time and accelerometers could both over- and under-estimate activPAL-derived sedentary time. For higher levels of average self-reported and accelerometer-derived sedentary time, self-reported total sitting time overestimated the accelerometer-derived measurement. This might be explained by those with high levels of sitting time also engaging in longer bouts of sitting time for which durations might be more difficult to estimate and more likely to be rounded up. Furthermore, these longer bouts might have been interrupted by non-sedentary activities which are registered by the accelerometers as non-sedentary, but which are included in the sedentary time reported by the participants.

Our validity findings differed between demographic subgroups. We observed stronger correlations and narrower limits of agreement for 65- to 74-year-old, male and tertiary educated participants compared to their counterparts. A first explanation for these differences in validity might be that the younger age group, men and those with tertiary education engage more frequently in sedentary behaviors that are easier to recall and report, such as car driving and computer use, compared to their respective counterparts. Furthermore, better cognitive functioning and capacity to recall and report past sedentary behaviors among 65–74 year old compared to 75+ year old participants might explain the better validity results in the younger age group. As mentioned, our validity results for men (rho = 0.35) were better compared to women (rho = 0.24). However, España-Romero et al. [37] reported similar correlations between self-reported sitting time and sedentary time measured by a combined heart rate and movement sensor among men (rho = 0.17) and women (rho = 0.18) in a sample of British 60- to 65-year-olds. The sample of España-Romero et al. [37] was younger than our sample and included many non-retired participants, which possibly means that these men and women were more likely to engage in similar sedentary behaviors (i.e. occupational sitting) and, hence, similar validity results. Our most substantial difference in correlation was found between non-tertiary (rho = 0.25) and tertiary educated participants (rho = 0.39). This is in line with findings by Sabia et al. [38] on the validity of self-reported physical activity among 60- to 83-year-old British participants. They found lower correlations between self-reported and accelerometer-derived PA among those with lower education or occupational position compared to participants with higher education or higher occupational position. Cerin et al. [19] reported better reliability of a question assessing overall sitting time among Hong Kong older adults living in high compared to low socio-economic neighborhoods. However, their validity results did not differ between high and low socio-economic neighborhoods. More research is needed to further examine demographic differences in validity and to increase the specificity of questionnaires for groups in which lower validity has been observed.

Overall, our findings on validity were not ideal; the correlation coefficients did not reach 0.50 (which was defined as good validity for physical activity questionnaires) [34] and the 95% limits of agreement were wide. In addition to the reasons described above, another possible explanation for this absence of strong validity is that the target period of our sedentary behavior questionnaire did not overlap with the period the accelerometer was worn. However, in our subsample for the reliability analysis both periods did overlap, but a correlation analyses between their self-reported total sitting time and accelerometer-derived sedentary time did result in a similar correlation (ρ = 0.32) and similar width of the 95% limits of agreement. Additionally, although we followed standard procedures for accelerometer initialization and processing, there is not yet a consensus about many of these procedures [27]. Although our findings showed only modest validity, they were no worse than those reported for previous questionnaires targeting older adults’ (specific) sedentary behavior(s) [15,16]. Moreover, our questionnaire targeted a wide range of specific sedentary behaviors which may have resulted in a lower level of underestimation of total sitting time compared to previous questionnaires [15,16]. Additionally, our questionnaire’s format is similar to the IPAQ, which might facilitate administration in studies that assess both physical activity and sedentary behaviors. Given the high prevalence of sedentary behaviors among older adults and the associated health risks, researchers should not delay studies on the health risks, prevalence and correlates of sedentary behaviors and could use the new questionnaire to assess older adults’ sedentary behaviors. Objective measures of sedentary time should be preferred, complemented with the questionnaire to get context-specific information. Our questionnaire might be especially useful for the specific sedentary behaviors for which we found acceptable reliability; TV viewing time, computer use, and car driving. In the meantime, more research is needed to develop questionnaires and objective criterion measures that measure older adults’ engagement in sedentary behaviors more accurately. These validity studies should use different criterion measures (e.g. Actigraph accelerometers and activPALs) and could include log books to examine the validity of specific sedentary behaviors.

Our newly-developed questionnaire for assessing multiple specific sedentary behaviors in older adults was found to be reasonably reliable for total sitting time. In contrast, only three of the twelve specific sedentary behaviors appeared to have acceptable test-retest reliability (i.e. TV viewing time, computer use, and car driving). Test-retest reliability for self-reported total sitting time in the current questionnaire appeared to be as good as or better than previous studies in older adults [15] and adults [9] using a sum of multiple specific sedentary behaviors and similar periods between test and retest. Similar to these previous studies, good reliability was found for TV viewing time. This might be explained by TV viewing being easy to recall accurately since it occurs on a regular basis, for prolonged periods, and at specific time points. The same explanation might be true for computer use, which was also found to have acceptable reliability in the current and previous studies [15,16]. However, in contrast to poor reliability found in previous studies [15,16], we found acceptable reliability for driving a car. Possibly, in our sample, being a car driver is connected to a distinct activity that is performed regularly by the older adults (e.g. going to the supermarket, visiting family) and is, therefore, more-readily recalled.

The remaining questions addressing specific sedentary behaviors did not demonstrate acceptable test-retest reliability, although they could be expected to occur at regular time points for relatively constant durations (e.g. sitting during meals). There might be several reasons for this absence of acceptable reliability. First, it might actually be difficult for older adults to recall and accurately estimate the duration of specific sedentary behaviors. Questionnaires assessing sedentary behavior(s) performed during the past day might offer a solution to this issue [39], however, these might be less accurate in capturing usual engagement in sedentary behavior(s). Secondly, our test and retest assessment of the specific sedentary behaviors did not target the same seven days. Consequently, the absence of acceptable reliability might simply reflect between-week variability in the sedentary behaviors. Despite the absence of acceptable reliability for the majority of the specific sedentary behavior items, we did find acceptable reliability for total sitting time. This might indicate that the total amount of sitting time does not vary substantially from week to week, but that how it is accumulated changes (one specific sedentary behavior might be replaced by another). Hence, the inclusion of all relevant sedentary behaviors might explain the good reliability results for our measure of self-reported total sitting time.

It should be noted that we tested an interview-based version of our sedentary behavior questionnaire and that our results may not be applicable to self-completion of the questionnaire. For a questionnaire assessing older adults’ physical activity, Dinger et al. [40] concluded that their observations of very good test-retest reliability (ICC = 0.91) might have resulted from the use of interviews rather than self-completion. Washburn et al. [41] found better validity results for a telephone-based physical activity questionnaire compared to a self-completion version, but the latter resulted in better test-retest reliability results. Furthermore, we used ‘the last seven days’ as the time frame to report sedentary behaviors. Among adults, similar reliability and validity results for self-reported total sitting time have been observed for ‘the last seven days’ and ‘the usual week’ time frame [23]. However, it has been argued that older adults might consider a usual rather than the last week when reporting their engagement in physical activity behaviors although they were asked to consider only the last week [42]. Therefore, in the current study, researchers responsible for data collection were explicitly trained to ensure that participants’ self-reports reflected engagement in sedentary behaviors during the last seven days. To our knowledge, no studies have investigated the influence of administration mode or time frame on the psychometrics of a sedentary behavior questionnaire among older adults. More research is necessary to determine the optimal mode of administration and time frame.

A first strength of the current study is the examination of a sedentary behavior questionnaire that included an extensive list of specific sedentary behaviors. Secondly, our questionnaire had a similar format as the IPAQ, which we used to increase the ease of administration (since the participants were acquainted with the format by previously completing the IPAQ). Thirdly, we investigated differences in validity according to age, gender and education. Our study has limitations, however. First is the use of accelerometers as criterion measure to assess sedentary behavior. Secondly, we only examined the validity of self-reported total sitting time and not the validity of self-reported specific sedentary behaviors. Future studies could include sedentary behavior log books to assess the validity of self-reported specific sedentary behaviors. Thirdly, older adults who were limited by their health to walk a couple of 100 meters were excluded from the current study. Therefore, our findings could not be generalized to older adults with such mobility impairment. Since mobility-impaired older adults may be at increased risk for high levels of sedentary time, future research should investigate the measurement properties of sedentary behavior questionnaires in this subgroup. Fourthly, the size of the subsample for our reliability analysis was not sufficient to perform reliability analyses in different demographic subgroups. The subsample for our reliability analysis was also rather highly educated which might have led to better reliability results.

To conclude, we examined criterion validity and test-retest reliability of a newly-developed sedentary behavior questionnaire, but our findings did not exhibit ideal validity for self-reported total sitting and test-retest reliability for most of the specific sedentary behaviors. However, our findings were comparable to what has been reported by previous studies. Furthermore, our questionnaire tended to result in a lower level of underestimation of sedentary time compared to other questionnaires, possibly explained by the inclusion of additional specific sedentary behaviors. We also observed better validity results for 65- to 74-year-old, male and tertiary educated participants compared to their counterparts. Further research is needed to develop self-report tools and objective criterion measures that accurately measure older adults’ engagement in specific sedentary behaviors and total sitting time.

## Competing interests

The authors declare that they have no competing interests.

## Authors’ contributions

JVC, VVH, IDB and BD designed the study. JVC and VVH led the data collection. JVC performed the statistical analyses and drafted the manuscript. All authors critically revised the content of the manuscript and have given final approval for publication.

## Pre-publication history

The pre-publication history for this paper can be accessed here:

http://www.biomedcentral.com/1471-2458/14/734/prepub

## Supplementary Material

Additional file 1 English version of the questionnaire assessing 12 particular sedentary behaviors. Click here for file

## References

[B1] Owen N Healy GN Matthews CE Dunstan DW Too much sitting: the population health science of sedentary behavior Exerc Sport Sci Rev 2010 38 105 113 20577058 10.1097/JES.0b013e3181e373a2 PMC3404815

[B2] Thorp AA Owen N Neuhaus M Dunstan DW Sedentary behaviors and subsequent health outcomes in adults a systematic review of longitudinal studies, 1996–2011 Am J Prev Med 2011 41 207 215 21767729 10.1016/j.amepre.2011.05.004

[B3] Grontved A Hu FB Television viewing and risk of type 2 diabetes, cardiovascular disease, and all-cause mortality a meta-analysis J Am Med Assoc 2011 305 2448 2455 10.1001/jama.2011.812 PMC4324728 21673296

[B4] Santos DA Silva AM Baptista F Santos R Vale S Mota J Sardinha LB Sedentary behavior and physical activity are independently related to functional fitness in older adults Exp Gerontol 2012 47 908 912 22884978 10.1016/j.exger.2012.07.011

[B5] Stamatakis E Davis M Stathi A Hamer M Associations between multiple indicators of objectively-measured and self-reported sedentary behaviour and cardiometabolic risk in older adults Prev Med 2012 54 82 87 22057055 10.1016/j.ypmed.2011.10.009

[B6] van der Ploeg HP Chey T Korda RJ Banks E Bauman A Sitting time and all-cause mortality risk in 222 497 Australian adults Arch Intern Med 2012 172 494 500 22450936 10.1001/archinternmed.2011.2174

[B7] Patel AV Bernstein L Deka A Feigelson HS Campbell PT Gapstur SM Colditz GA Thun MJ Leisure time spent sitting in relation to total mortality in a prospective cohort of US adults Am J Epidemiol 2010 172 419 429 20650954 10.1093/aje/kwq155 PMC3590043

[B8] Gardiner PA Healy GN Eakin EG Clark BK Dunstan DW Shaw JE Zimmet PZ Owen N Associations between television viewing time and overall sitting time with the metabolic syndrome in older men and women: the Australian Diabetes Obesity and Lifestyle study J Am Geriatr Soc 2011 59 788 796 21568949 10.1111/j.1532-5415.2011.03390.x

[B9] Healy GN Clark BK Winkler EAH Gardiner PA Brown WJ Matthews CE Measurement of adults’ sedentary time in population-based studies Am J Prev Med 2011 41 216 227 21767730 10.1016/j.amepre.2011.05.005 PMC3179387

[B10] Hamer M Stamatakis E Screen-based sedentary behavior, physical activity, and muscle strength in the English longitudinal study of ageing Plos One 2013 8 1 5 10.1371/journal.pone.0066222 PMC3670922 23755302

[B11] Kesse-Guyot E Charreire H Andreeva VA Touvier M Hercberg S Galan P Oppert J-M Cross-sectional and longitudinal associations of different sedentary behaviors with cognitive performance in older adults Plos One 2012 7 1 8 10.1371/journal.pone.0047831 PMC3474738 23082222

[B12] Owen N Sugiyama T Eakin EE Gardiner PA Tremblay MS Sallis JF Adults’ sedentary behavior determinants and interventions Am J Prev Med 2011 41 189 196 21767727 10.1016/j.amepre.2011.05.013

[B13] Atkin AJ Gorely T Clemes SA Yates T Edwardson C Brage S Salmon J Marshall SJ Biddle SJH Methods of measurement in epidemiology: sedentary behaviour Int J Epidemiol 2012 41 1460 1471 23045206 10.1093/ije/dys118 PMC3465769

[B14] Clark BK Sugiyama T Healy GN Salmon J Dunstan DW Owen N Validity and reliability of measures of television viewing time and other non-occupational sedentary behaviour of adults: a review Obes Rev 2009 10 7 16 18631161 10.1111/j.1467-789X.2008.00508.x

[B15] Gardiner P Clark BK Healy GN Eakin EG Winkler EAH Owen N Measuring older adults’ sedentary time: reliability, validity, and responsiveness Med Sci Sports Exerc 2011 43 2127 2133 21448077 10.1249/MSS.0b013e31821b94f7

[B16] Hekler EB Buman MP Haskell WL Conway TL Cain KL Sallis JF Saelens BE Frank LD Kerr J King AC Reliability and validity of CHAMPS self-reported sedentary-to-vigorous intensity physical activity in older adults J Phys Act Health 2012 9 225 236 22368222 10.1123/jpah.9.2.225 PMC4733646

[B17] Rosenberg DE Norman GJ Wagner N Patrick K Calfas KJ Sallis JF Reliability and validity of the Sedentary Behavior Questionnaire (SBQ) for adults J Phys Act Health 2010 7 697 705 21088299 10.1123/jpah.7.6.697

[B18] Rey-Lopez JP Ruiz JR Ortega FB Verloigne M Vicente-Rodriguez G Gracia-Marco L Gottrand F Molnar D Widhalm K Zaccaria M Cuenca-Garcia M Sjostrom M De Bourdeaudhuij I Moreno LA Reliability and validity of a screen time-based sedentary behaviour questionnaire for adolescents: the HELENA study Eur J Pub Health 2012 22 373 377 21498560 10.1093/eurpub/ckr040

[B19] Cerin E Barnett A Cheung MC Sit CHP Macfarlane DJ Chan WM Reliability and validity of the IPAQ-L in a sample of Hong Kong urban older adults: does neighborhood of residence matter? J Aging Phys Act 2012 20 402 420 22186607 10.1123/japa.20.4.402

[B20] Ware J Kosinski M Keller S SF-36 Physical and mental health summary scales: a user manual and interpretation guide 1994 Boston: The Health Institute, New England Medical Center

[B21] Haywood KL Garratt AM Fitzpatrick R Quality of life in older people: a structured review of generic self-assessed health instruments Qual Life Res 2005 14 1651 1668 16119178 10.1007/s11136-005-1743-0

[B22] Helmerhorst HJF Brage S Warren J Besson H Ekelund U A systematic review of reliability and objective criterion-related validity of physical activity questionnaires Int J Behav Nutr Phys Act 2012 9 1 55 22938557 10.1186/1479-5868-9-103 PMC3492158

[B23] Craig CL Marshall AL Sjostrom M Bauman AE Booth ML Ainsworth BE Pratt M Ekelund U Yngve A Sallis JF Oja P International physical activity questionnaire: 12-country reliability and validity Med Sci Sports Exerc 2003 35 1381 1395 12900694 10.1249/01.MSS.0000078924.61453.FB

[B24] Forsen L Loland NW Vuillemin A Chinapaw MJM van Poppel MNM Mokkink LB van Mechelen W Terwee CB Self-administered physical activity questionnaires for the elderly a systematic review of measurement properties Sports Med 2010 40 601 623 20545382 10.2165/11531350-000000000-00000

[B25] Salmon J Owen N Crawford D Bauman A Sallis JF Physical activity and sedentary behavior: a population-based study of barriers, enjoyment, and preference Health Psychol 2003 22 178 188 12683738 10.1037//0278-6133.22.2.178

[B26] Matton L Wijndaele K Duvigneaud N Duquet W Philippaerts R Thomis M Lefevre J Reliability and validity of the Flemish physical activity computerized questionnaire in adlults Res Q Exerc Sport 2007 78 293 306 17941534 10.1080/02701367.2007.10599427

[B27] Taraldsen K Chastin SFM Riphagen II Vereijken B Helbostad JL Physical activity monitoring by use of accelerometer-based body-worn sensors in older adults: a systematic literature review of current knowledge and applications Maturitas 2012 71 13 19 22134002 10.1016/j.maturitas.2011.11.003

[B28] Freedson P Bowles HR Troiano R Haskell W Assessment of physical activity using wearable monitors: recommendations for monitor calibration and use in the field Med Sci Sports Exerc 2012 44 S1 S4 22157769 10.1249/MSS.0b013e3182399b7e PMC3245520

[B29] Grant PM Ryan CG Tigbe WW Granat MH The validation of a novel activity monitor in the measurement of posture and motion during everyday activities Br J Sports Med 2006 40 992 997 16980531 10.1136/bjsm.2006.030262 PMC2577473

[B30] Choi L Ward SC Schnelle JF Buchowski MS Assessment of wear/nonwear time classification algorithms for triaxial accelerometer Med Sci Sports Exerc 2012 44 2009 2016 22525772 10.1249/MSS.0b013e318258cb36 PMC3443532

[B31] Grimm EK Swartz AM Hart T Miller NE Strath SJ Comparison of the IPAQ-Short Form and accelerometry predictions of physical activity in older adults J Aging Phys Act 2012 20 64 79 22190120 10.1123/japa.20.1.64

[B32] Evenson KR Buchner DM Morland KB Objective measurement of physical activity and sedentary behavior among US adults aged 60 years or older Prev Chronic Dis 2012 9 1 10 PMC3277387 22172193

[B33] Freedson PS Melanson E Sirard J Calibration of the Computer Science and Applications, Inc. accelerometer Med Sci Sports Exerc 1998 30 777 781 9588623 10.1097/00005768-199805000-00021

[B34] Terwee CB Mokkink LB van Poppel MNM Chinapaw MJM van Mechelen W de Vet HCW Qualitative attributes and measurement properties of physical activity questionnaires a checklist Sports Med 2010 40 525 537 20545379 10.2165/11531370-000000000-00000

[B35] Bland JM Altman DG Measuring agreement in method comparison studies Stat Methods Med Res 1999 8 135 160 10501650 10.1177/096228029900800204

[B36] Aaronson N Alonso J Burnam A Lohr KN Patrick DL Perrin E Stein REK Sci Advisory Comm Med Outcomes T Assessing health status and quality-of-life instruments: attributes and review criteria Qual Life Res 2002 11 193 205 12074258 10.1023/a:1015291021312

[B37] Espana-Romero V Golubic R Martin KR Hardy R Ekelund U Kuh D Wareham NJ Cooper R Brage S Teams NSDC Comparison of the EPIC Physical Activity Questionnaire with combined heart rate and m ovement sensing in a nationally representative sample of older British adults Plos One 2014 9 1 10 10.1371/journal.pone.0087085 PMC3916297 24516543

[B38] Sabia S van Hees VT Shipley MJ Trenell MI Hagger-Johnson G Elbaz A Kivimaki M Singh-Manoux A Association between questionnaire- and accelerometer-assessed physical activity: the role of sociodemographic factors Am J Epidemiol 2014 179 781 790 24500862 10.1093/aje/kwt330 PMC3939851

[B39] Clark BK Winkler E Healy GN Gardiner PG Dunstan DW Owen N Reeves MM Adults’ past-day recall of sedentary time: reliability, validity, and responsiveness Med Sci Sports Exerc 2013 45 1198 1207 23274615 10.1249/MSS.0b013e3182837f57

[B40] Dinger MK Oman F Taylor EL Vesely SK Able J Stability and convergent validity of the Physical Activity Scale for the Elderly (PASE) J Sports Med Phys Fit 2004 44 186 192 15470317

[B41] Washburn RA Smith KW Jette AM Janney CA The Physical Activity Scale for the Elderly (PASE) - development and evaluation J Clin Epidemiol 1993 46 153 162 8437031 10.1016/0895-4356(93)90053-4

[B42] Heesch KC van Uffelen JGZ Hill RL Brown WJ What do IPAQ questions mean to older adults? Lessons from cognitive interviews Int J Behav Nutr Phys Act 2010 7 35 20459758 10.1186/1479-5868-7-35 PMC3224924

