# Supplementary material for: Older adults’ reporting of specific sedentary behaviors: validity and reliability
Source: BMC Public Health. 2014 Jul 21;14:734. doi: 10.1186/1471-2458-14-734 (PMC4223385; doi:10.1186/1471-2458-14-734)
Supplement: Additional file 1 — English version of the questionnaire assessing 12 particular sedentary behaviors. [file 1471-2458-14-734-S1.pdf]

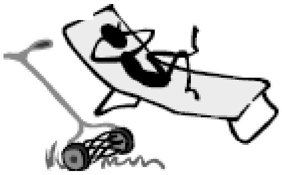

<u>Time spent sitting</u>

The following questions are about the time you spent sitting at work, at home, while studying or during leisure in the last 7 days. This includes time you sat behind a desk, received visitors, reading, viewing television, and during motorized transport.

1a **During the last seven days, on how many days did you use public transport (train, bus or tram)?**

________ days per week

O No *(skip to question 1c)*

1b **How much time did you usually spend on one of those days using public transport?**

____ hours ___ minutes per day

1c **During the last seven days, on how many days were you the driver of a motorized vehicle (e.g. moped, moto, car)?**

________ days per week

O No *(skip to question 1e)*

1d **How much time did you usually spend on one of those days driving a motorized vehicle?**

____ hours ___ minutes per day

1e **During the last seven days, on how many days were you a passenger in a motorized vehicle?**

________ days per week

O No *(skip to question 1g)*

1f **How much time did you usually spend on one of those days being a passenger in a motorized vehicle?**

____ hours ___ minutes per day

1g **During the last seven days, on how many days did you use a computer?**

________ days per week

O No *(skip to question 1i)*

1h **How much time did you usually spend on one of those days using a computer?**

____ hours ___ minutes per day

1i **During the last seven days, on how many days did you read (book, newspaper, magazine)?**

________ days per week

O No *(skip to question 1k)*

1j How much time did you usually spend on one of those days reading?

____ hours ___ minutes per day

1k During the last seven days, on how many days did you watch television?

________ days per week

O No *(skip to question 1m)*

1l How much time did you usually spend on one of those days watching television?

____ hours ___ minutes per day

1m During the last seven days, on how many days did you engage in sitting hobbies such as handicraft, playing cards, etc.?

________ days per week

O No *(skip to question 1o)*

1n How much time did you usually spend on one of those days in sitting hobbies?

____ hours ___ minutes per day

1o During the last seven days, on how many days did you make a phone call while sitting?

________ days per week

O No *(skip to question 1q)*

1p **How much time did you usually spend on one of those days making a phone call while sitting?**

____ hours ___ minutes per day

1q **During the last seven days, on how many days did you chat with friends or acquaintances (not on the phone) or listened to music while sitting?**

________ days per week

O No *(skip to question 1s)*

1r **How much time did you usually spend on one of those days chatting with friends or acquaintances (not on the phone) or listening to music while sitting?**

____ hours ___ minutes per day

1s **During the last seven days, on how many days did you do household chores, such as ironing, folding laundry or preparing a meal, while sitting?**

________ days per week

O No *(skip to question 1u)*

1t **How much time did you usually spend on one of those days doing household chores while sitting?**

____ hours ___ minutes per day

1u During the last seven days, how much time did you usually spend per day eating while sitting?

____ hours ___ minutes per day
